# Use of catch-up vaccinations in the second year of life (2YL) platform to close immunity gaps: A secondary DHS analysis in Pakistan, Philippines, and South Africa

**DOI:** 10.1016/j.vaccine.2022.10.040

**Published:** 2023-01-04

**Authors:** Porcia Manandhar, Kathleen Wannemuehler, M. Carolina Danovaro-Holliday, Laura Nic Lochlainn, Stephanie Shendale, Samir V. Sodha

**Affiliations:** a Department of International Health, Johns Hopkins Bloomberg School of Public Health, Baltimore, MD, USA; b Department of Biostatistics & Medical Informatics, University of Wisconsin – Madison, WI, USA; c World Health Organization, Geneva, Switzerland

**Keywords:** Immunization, Life course, Catch-up vaccination, Vaccination timeliness, Demographic and Health Survey (DHS), Missed opportunities for vaccination (MOV)

## Abstract

**Background:**

Immunity gaps caused by COVID-19-related disruptions highlight the importance of catch-up vaccination. Number of countries offering vaccines in second year of life (2YL) has increased, but use of 2YL for catch-up vaccination has been variable. We assessed pre-pandemic use of 2YL for catch-up vaccination in three countries (Pakistan, the Philippines, and South Africa), based on existence of a 2YL platform (demonstrated by offering second dose of measles-containing vaccine (MCV2) in 2YL), proportion of card availability, and geographical variety.

**Methods:**

We conducted a secondary data analysis of immunization data from Demographic and Health Surveys (DHS) in Pakistan (2017–2018), the Philippines (2017), and South Africa (2016). We conducted time-to-event analyses for pentavalent vaccine (diphtheria-tetanus-pertussis-Hepatitis B-*Haemophilus influenzae* type b [Hib]) and MCV and calculated use of 2YL and MCV visits for catch-up vaccination.

**Results:**

Among 24–35-month-olds with documented dates, coverage of third dose of pentavalent vaccine increased in 2YL by 2%, 3%, and 1% in Pakistan, Philippines, and South Africa, respectively. MCV1 coverage increased in 2YL by 5% in Pakistan, 10% in the Philippines, and 3% in South Africa. In Pakistan, among 124 children eligible for catch-up vaccination of pentavalent vaccine at time of a documented MCV visit, 45% received a catch-up dose. In the Philippines, among 381 eligible children, 38% received a pentavalent dose during an MCV visit. In South Africa, 50 children were eligible for a pentavalent vaccine dose before their MCV1 visit, but only 20% received it; none with MCV2.

**Conclusion:**

Small to modest vaccine coverage improvements occurred in all three countries through catch-up vaccination in 2YL but many missed opportunities for vaccination continue to occur. Using the 2YL platform can increase coverage and close immunity gaps, but immunization programmes need to change policies, practices, and monitor catch-up vaccination to maximize the potential.

## 1 Introduction

Although vaccination is a proven cost-effective preventative health intervention, childhood routine immunization coverage, based on the third dose of the diphtheria-tetanus-pertussis (DTP3) vaccine, remained relatively stagnant in the last decade (2010–2019) before the SARS-CoV-2 (COVID-19) pandemic [1]. Immunization service disruptions from the pandemic led to an estimated 5% drop in global DTP3 coverage from 86% in 2019 to 81% in 2021 [2], affecting multiple countries across all regions [3], [4], [5], [6], [7]. Resulting immunity gaps amongst children worldwide increase the risk of vaccine-preventable disease (VPDs) outbreaks and catch-up vaccination activities have become a global priority to close immunity gaps [8].

When the Expanded Programme on Immunization (EPI) was initially established based on the smallpox eradication program in 1974, the focus was on vaccination during the first year of life with four vaccines: Bacillus Calmette–Guérin (BCG), Diphtheria-Tetanus- Pertussis (DTP), poliomyelitis vaccine, and measles-containing vaccine (MCV) in addition to smallpox [9]. But EPI has evolved to recommend vaccines and doses across the life course, including during the second year of life (2YL). The 2YL platform for vaccination has become increasingly established in countries through the introduction of the second dose of measles-containing vaccine (MCV2) and DTP booster doses among others. A well-functioning 2YL platform for vaccination can expand opportunities for catch-up vaccination for vaccine doses missed in infancy. Optimal use of the 2YL platform for catch-up vaccination can play a critical role in reducing immunity gaps, including those created by the COVID-19 pandemic.

Catch-up vaccination of routine vaccines can be provided through traditional routine immunization delivery platforms, such as scheduled fixed site or outreach immunization sessions, or campaign-like events known as periodic intensification of routine immunization (PIRI). Although PIRIs can rapidly catch-up children, they are resource-intensive and may not be feasible to implement for all populations, especially during times of fiscal constraint. Hence, countries will need to increasingly rely on improving the ability of traditional routine immunization delivery platforms to catch-up children through every opportunity for vaccination and reduce missed opportunities for vaccination (MOV) [10], [11].

We aimed to quantitatively assess the pre-pandemic use of the 2YL platform for catch-up vaccination of first-year vaccine doses using the Demographic and Health Surveys (DHS) data from Pakistan, the Philippines, and South Africa.

## 2 Methods

### 2.1 Country selection

We selected a country from three of the six World Health Organization (WHO) regions based on three criteria: i) having a DHS survey conducted within five years of analysis time, ii) having at least 50% of vaccination cards (also known as a home-based record (HBR)) seen amongst the 12–23-month cohort, and iii) having an established 2YL platform based on MCV2 in the immunization schedule for at least five years before the DHS. The three countries that met the criteria were Pakistan (DHS 2017–2018), the Philippines (2017), and South Africa (2016) [12], [13], [14]. The introduction of MCV2 to routine immunization schedules establishes 2YL platforms which took place in 1994 for South Africa and in 2009 for Pakistan and the Philippines (Table 1). Their routine immunization schedules can be found in Table 1.

**Table 1:** Routine Immunization Schedule in Pakistan, Philippines, and South Africa.

**Country**	**EPI Schedule**					
	**Penta 1**	**Penta 2**	**Penta 3**	**Penta 4**	**MCV1**	**MCV2 (year of introduction)**
Pakistan	6 weeks	10 weeks	14 weeks	–	9 months	15 months (2009)
Philippines	6 weeks	10 weeks	14 weeks	–	9 months	12 months (2009)
South Africa*	6 weeks	10 weeks	14 weeks	18 months	6 months	12 months (1994)

* For South Africa, a schedule change occurred on December 01, 2015. Penta (DTaP-IPV-Hib) was replaced by hexavalent (Penta + HepB); the two-dose MCV schedule changed from 39 and 78 weeks to 26 and 52 weeks, respectively. The children who were 12–35 months at the time of the survey would have received their doses under the old schedule. Penta for Pakistan and the Philippines refers to DTP-HepB-Hib, while for South Africa it was DTaP-inactivated polio vaccine (IPV)-Hib.

### 2.2 Data management and analysis

We conducted a secondary data analysis of vaccination data from children aged 12–35 months whose mothers participated in a DHS. The data management and analysis for this study were completed in R v4.0 [15].

The DHS dataset source was the child recode files for each country. Details on the surveys can be obtained from the publicly available reports [12], [13], [14]. Details on the DHS recode files can be found in the DHS recode manual [4] and map [5]. In brief, each of these surveys is a nationally representative stratified cluster survey. Households are selected randomly; women 15–49 years are eligible to participate. Vaccination information is collected on surviving children under 3 years of age whose mothers are members of a selected household. The Pakistan survey had 16 sampling strata and 580 primary sampling units (PSUs). The Pakistan DHS final report, and this current study, exclude the areas of Gilgit Baltistan, Azad Jammu and Kashmir. The Philippines survey had 117 sampling strata and 1250 PSUs. The South Africa survey was comprised of 26 sampling strata and 750 PSUs.

If a HBR was seen at the time of the survey, the date components (month, day, year) were captured for each antigen in separate fields. Each date component coded 97 (inconsistent value), 98 (response: Don’t know), and 99 (response: missing) were set to missing prior to calculating the date of vaccination. For a given dose, if month and year were available and day was missing, we imputed the day to be the 15th of the month. From the available modified date components, the vaccine date for the respective dose was created. For each child, an antigen dose was classified as being documented with a vaccination date, documented with a vaccination card (with a date or other marking on the card), by caregiver recall, or not vaccinated. When calculating coverage for the vaccine-dose series, (*e.g.* the 3 doses of Pentavalent DTP-*Haemophilus influenzae* type b [Hib]-HepB (Penta), or the 2 doses of measles containing vaccine (MCV)), the data on receipt of vaccine by vaccination card + recall was modified to ensure that doses were sequential. For example, if the original data indicated the child received Penta 1 and Penta 3 by vaccination card or recall but not Penta 2, the information on Penta 3 was transferred to the Penta 2 variables (vaccination status and date), and the information on Penta 3 was deleted as it is done by DHS for their survey reports.

Data analysis included vaccination coverage, drop-out percentages, and time to event (vaccination) using Kaplan-Meier curves. Vaccination coverage percentages are presented in three ways: documented by date, documented by card, and documented by either card or recall (card + recall). These coverage estimates and 95% CIs (logit interval) were calculated accounting for the survey design. A descriptive analysis of time to vaccination documented with a date for the three Penta and two MCV doses is presented as reverse Kaplan-Meier curves calculated using the R *survival* package [16] without accounting for the survey design because we did these analyses only for a sub-sample: the time of vaccination is only captured on those children with a documented date of birth and date of vaccination. In this time-to-event analysis, those classified as vaccinated by documentation without a date, caregiver recall, or unvaccinated were classified as not having the event of interest. These children’s follow-up time was censored at the time of the survey. Further exploration of catch-up doses for the Penta series at the MCV1 or MCV2 visits are presented descriptively. This Kaplan-Meier analysis was limited to children aged 12–35-months who had at least one documented dose of MCV. All figures were created using the R *ggplot2* package [17].

## 3 Ethics

Ethical approval for the conduct of the surveys was obtained by the institutions involved in data collection. All data used were obtained anonymized through DHS and then used in this secondary analysis.

## 4 Results

### 4.1 Documentation of vaccination

The Pakistan survey enrolled 1893 children in 12–23 months cohort and 1974 children in the 24–35 months cohort. In the Philippines, there were 1986 and 2015 children and in South Africa, there were respectively 670 and 680 children respectively in 12–23 months and the 24–35 months age cohort (Table 2).

**Table 2:** Demographic Health Surveys (DHS), Routine immunization/child health card availability.

**Country**	**Age**	**Children**	**Reported having a card for the child**	**Card was seen at survey**	**Card had at least one vaccination date**
	**(months)**	**N**	**n (%)**	**n (%)**	**n (%)**
Pakistan (DHS 2017–2018)	12–23	1893	1483 (78)	1071 (57)	1066 (56)
	24–35	1974	1468 (74)	806 (41)	801 (41)
Philippines (DHS 2017)	12–23	1986	1872 (94)	1301 (66)	1297 (65)
	24–35	2015	1854 (92)	1053 (52)	1046 (52)
South Africa (DHS 2016)	12–23	670	654 (98)	473 (71)	348 (52)
	24–35	676	663 (98)	415 (61)	332 (49)

The percentage of children who reportedly ever had a vaccination card was 75% in Pakistan, 92% in the Philippines, and 98% in South Africa. The percentage of children with a card seen by surveyors was lowest in Pakistan (58% in 0–11 months; 57% in 12–23 months and 41% in 24–35 months), followed by the Philippines (74% in 0–11 months; 66% in 12–23 months and 52% in 24–35 months). The percentage of children with a card seen in South Africa was 80% in 0–11-month-olds, 71% in 12–23-month-olds, and 61% in 24–35-month-olds. For South Africa, 12–20% of the children had cards with doses documented but without a date, whereas in Pakistan and the Philippines this was observed in only 0.3% and 0.4% of the children in total respectively.

### 4.2 Coverage and Drop-out estimates

The estimated percentage of children with 3 doses of Penta by vaccination card + recall was 75% and 76% in the 12–23 and 24–35-month-olds in Pakistan; 80% and 72% in 12–23-month-olds and 24–35-month-olds in the Philippines, and 65% and 63% in 12–23 and 24–35-month-olds cohorts in South Africa respectively (Table 3). Vaccination card + recall coverage for the first dose of MCV (MCV1) in Pakistan among 12–23 and 24–35-month-olds was 73% and 75%, 80% and 81% in the Philippines, and 86% and 84% in South Africa respectively. The second dose of MCV(MCV2) in the 24–35-month-olds was 67% in Pakistan, 46% in the Philippines, and 59% in South Africa.

**Table 3:** Routine Immunization card documented and card + caregiver recall coverage.

**Country**	**Antigen**	**Coverage (95% CI)**^1^ **12**–**23 months**			**Coverage (95% CI)** **24**–**35 months**		
		**Date Documented**^2^	**Card Documented**^3^	**Card + Recall**	**Date Documented**^2^	**Card Documented**^3^	**Card + Recall**
Pakistan	Penta 1	61 (57, 64)	62 (58, 65)	86 (83, 89)	45 (41, 49)	46 (42, 50)	83 (79, 86)
	Penta 2	59 (55, 63)	60 (56, 64)	83 (80, 86)	44 (40, 48)	45 (41, 49)	80 (75, 83)
	Penta 3	56 (52, 60)	57 (53, 61)	75 (72, 79)	43 (39, 47)	44 (40, 48)	76 (71, 80)
	MCV1	52 (48, 56)	53 (49, 57)	73 (69, 77)	40 (37, 45)	41 (37, 45)	75 (71, 79)
	MCV2	N/A	N/A	N/A	35 (31, 39)	36 (32, 40)	67 (62, 71)
Philippines	Penta 1	62 (59, 66)	62 (59, 66)	87 (84, 88)	51 (47, 54)	51 (47, 54)	83 (80, 85)
	Penta 2	61 (58, 64)	61 (58, 64)	83 (80, 85)	49 (46, 53)	49 (46, 53)	76 (73, 79)
	Penta 3	59 (56, 63)	59 (56, 63)	80 (77, 82)	46 (43, 50)	46 (43, 50)	72 (69, 75)
	MCV1	57 (54, 61)	57 (54, 61)	80 (78, 83)	49 (46, 53)	49 (46, 53)	81 (78, 83)
	MCV2	N/A	N/A	N/A	33 (30, 36)	33 (30, 37)	46 (43, 50)
South Africa	Penta 1	47 (41, 52)	66 (61, 71)	91 (88, 94)	48 (42, 54)	61 (56, 66)	88 (84, 90)
	Penta 2	45 (40, 51)	64 (59, 69)	75 (71, 79)	47 (42, 53)	60 (55, 65)	71 (66, 76)
	Penta 3	43 (38, 48)	62 (56, 67)	65 (60, 70)	44 (39, 50)	57 (52, 62)	63 (58, 68)
	MCV1	43 (38, 48)	62 (57, 67)	86 (83, 89)	47 (41, 52)	58 (53, 63)	84 (80, 88)
	MCV2	N/A	N/A	N/A	37 (32, 43)	48 (43, 53)	59 (54, 64)

1 Weighted coverage, accounting for the stratified cluster design

2 Card documented by date dose was received

3 Card documented by date or other mark indicated dose was received

Based on these point estimates of coverage, the drop-out from Penta 1 to Penta 3 by vaccination card + recall was 13% and 8% for the 12–23-month-olds and 24–35-month-olds in Pakistan, respectively; 8% and 13% for the 12–23-month-olds and 24–35-month-olds in the Philippines, and 29% and 28% for the 12–23-month-olds and 24–35-month-olds in South Africa. For MCV, the percent drop-out from MCV1 to MCV2 among 24–35-month-olds was 11% in Pakistan, 43% in the Philippines, and 30% in South Africa.

### 4.3 2YL and Time to vaccination

Amongst 24–35-month-old children who have documented vaccination cards with dates, 557 (69.5%) in Pakistan, 757 (72.4%) in the Philippines, and 282 (84.9%) in South Africa utilized the 2YL platform by receiving at least one vaccine on or after their first birthday.

**Fig. 1:**
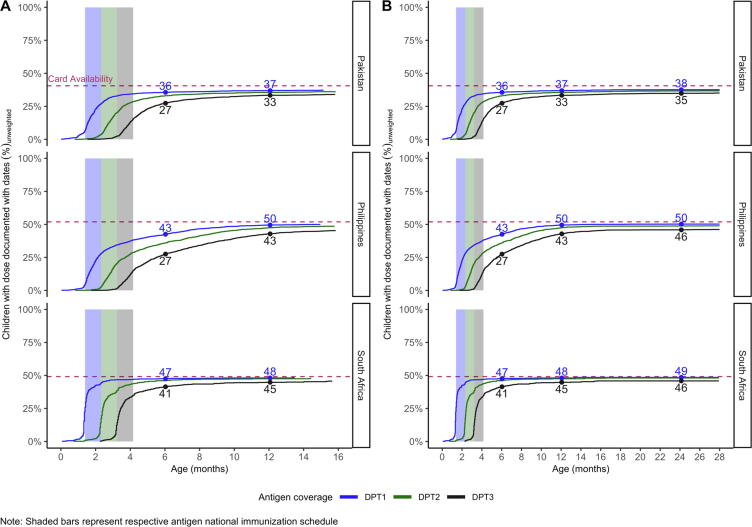
1A and 1B: Vaccination coverage by age in months of DTP amongst 12–23-months-olds (Figure A) and 24–35-month-olds (Figure B) in Pakistan (DHS 2017–2018), the Philippines (DHS 2017), and South Africa (DHS 2016).

The reverse Kaplan-Meier curves for the three-dose Penta series illustrate the drop-out from the first to third dose in 12–23-month-olds and 24–35-month-olds in all three countries (Fig. 1A, Fig. 1B). Larger delays in the receipt of the second and third Penta doses are observed in Pakistan and the Philippines compared with South Africa. However, both countries continue to provide Penta throughout the first year of life. The additional year of follow-up in the older cohort with documented vaccination cards with dates shows that a small percentage of children are receiving a dose of Penta in the second year of life. Among 24–35-months-olds, Penta 3 coverage from 12 months of age to 24 months increases by 2%, 3%, and 1% in Pakistan, the Philippines, and South Africa, respectively (Fig. 1B).

**Fig. 2:**
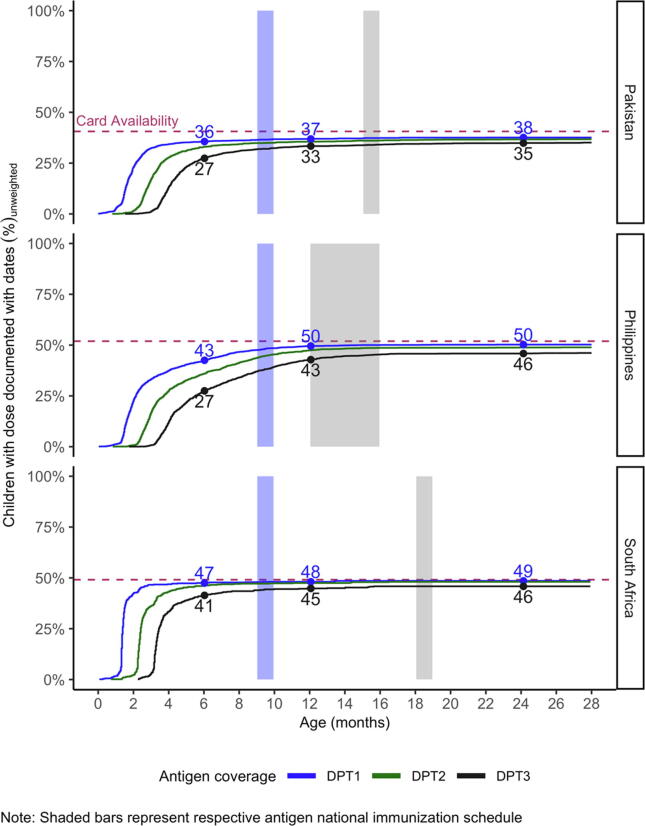
Vaccination coverage by age in months of MCV amongst 24–35 month-olds in Pakistan (DHS 2017–2018), the Philippines (DHS 2017), and South Africa (DHS 2016).

The unweighted percentage of children with a documented vaccination date for MCV1 by 12 months of age was 28% in Pakistan, 38% in the Philippines, and 43% in South Africa (Fig. 2). The increase in unweighted percentage of MCV1 from 12-months of age to 24-months is 5% in Pakistan, 10% in the Philippines, and 3% in South Africa. The Philippines’ figure shows a noticeable increase in uptake at 12 months, which coincides with the start of the recommended window for MCV2.

### 4.4 Catch-up vaccination of Penta with MCV doses

In Pakistan, among 1478 children with a date documented visit for MCV1 or MCV2, 124 (8%) did not have all three documented Penta doses (by dates or other markings) before the MCV1 visit (Table 4). Among these 124 children eligible for a Penta catch-up vaccination, only 56 (45%) received a catch-up Penta dose during an MCV visit (51 at MCV1 visits, one at the MCV2 visit in 2YL, and 4 during both MCV1 and MCV2 visits). While 21% received a Penta dose at a different time than the MCV visits, 34% still did not receive any catch-up Penta doses. In the Philippines, among 381 children with less than three documented Penta dose before the MCV1 visit, 146 (38%) received a Penta dose during an MCV visit (112 at MCV1 visits, 18 at MCV2 visits, and 16 at both MCV1 and MCV2 visits). An additional 35% received a Penta dose at a different date than the MCV visits, but 26% did not receive any Penta catch-up doses. In South Africa, 50 children had not completed the Penta series before the MCV1 visit and among them, 10 (20%) received Penta with the MCV1 visit but none with the MCV2 visit. While 26% of these children received another Penta dose on a different visit, 54% did not receive an additional Penta dose.

**Table 4:** Opportunity for Penta catch-up at MCV1 or MCV2 visit: 12–35 months.

				**Had < 3 documented**^1^**doses of Penta prior to the first MCV visit**			
**Country**	**Card Seen**	**MCV1 or MCV2 documented by a date**	**Had 3 documented**^1^**doses Penta prior to first MCV**	**Total** **N**	**Received with MCV** **n(%)**	**Received on a different date than MCV1 or MCV2** **n(%)**	**Did not have another date documented Penta dose** **n(%)**
Pakistan	1877	1478	1354	124	56 (45) 51 w/ MCV1 1 w/ MCV2 4 w/ both^3^	26 (21)	42 (34)
Philippines	2354	2128	1747	381	146 (38) 112 w/MCV1 18 w/ MCV2 16 w/ both^3^	135 (35)	100 (26)
South Africa^2^	888	633	583	50	10 (20) 10 w/ MCV1	13 (26)	27 (54)

1 documented by date or by other mark on the vaccination card.

2 South Africa has a 4th dose of Penta scheduled at the same time as MCV2. The information on this dose, captured separately in the data collection form, was not used in this analysis.

3 both means that children received a DPT with MCV1 and a DPT with MCV2.

## 5 Discussion

The strength of 2YL platforms for catch-up vaccination has not been systematically assessed to date. Our analysis shows small to modest improvements in coverage in all three countries due to catch-up vaccination in the 2YL, but there were still many opportunities for catch-up vaccination that were missed. These missed opportunities highlight the need to improve catch-up vaccination through enhanced functionality of existing routine immunization programmes, particularly through strengthening the relatively under-utilized 2YL platform.

Our results suggest that catch-up vaccination practices were suboptimal in the three countries assessed. Among under-vaccinated children who demonstrated continued access and utilization of immunization services, large proportions (range: 26–54%) in all three countries did not receive any of the catch-up Pentavalent vaccine doses for which they were eligible during an MCV visit. Our analysis is based on pre-COVID-19 pandemic data but the ability of countries to provide catch-up vaccinations is critically more important now due to the routine immunization disruptions caused by the COVID-19 pandemic and the subsequent reductions in routine childhood vaccinations across the globe [18], [19], [20], [21]. VPDs, such as measles which had been surging in many countries before the COVID-19 pandemic [22], [23], [24], [25], [26], [27], will be at an even higher risk for outbreaks because of the increased immunity gaps. This applies to poliomyelitis, meningococcal disease in the meningitis belt, and other outbreak-prone diseases as well. Low and lower-middle-income countries which already had low baseline coverage and comparatively weaker health systems before the pandemic, will be particularly vulnerable to increased morbidity and mortality from VPD outbreaks.

A robust 2YL platform capable of catching up children on first-year antigens during 2YL can contribute to a successful catch-up vaccination strategy and to reduce drop-out rates. Our analysis demonstrated that the 2YL platform can be used to provide catch-up vaccinations for MCV1 in the three assessed countries, but only did so at small to moderate levels (3–10% improvement during 2YL). The number of countries with a 2YL platform has dramatically increased in recent years, particularly with the acceleration of MCV2 introduction; countries recommending MCV2 has increased from 95 in 2000 to 179 in 2020 [28]. The 2YL platform will become more important as additional vaccines (*e.g.* DTP booster doses, malaria vaccine, meningitis vaccines) with vaccination schedules for children over 1 year of age become increasingly introduced in low and lower-and-middle-income countries [29]. The 2YL platform also creates opportunities for integration to maximize services in settings with limited resources by improving access and delivery of other child survival interventions, such as nutrition, growth monitoring, and deworming [30].

Missed opportunities for vaccination (MOVs) are an important impediment to providing catch-up vaccination through routine immunization. A MOV refers to any contact with health services by an individual (child or person of any age) who is eligible for vaccination (*e.g.* unvaccinated or partially vaccinated and free from any contraindications to vaccination), but does not result in the person receiving one or more of the vaccine doses for which he or she is eligible [31]. Our analysis showed concerning MOV occurring in all three countries with other vaccination visits. The prevalence of MOV can vary by setting, and a pooled MOV prevalence from 41 studies found one in three children experienced a MOV (32.2%; 95% CI: 26.8–37.7). More recent MOV country assessments have found the percentage of MOV to be as high as 76% in some settings [32], [33], [34], [35]. Despite having contact with health facilities for vaccination, sick visits, and vitamin A supplementation, MOVs for first-year antigens continue to occur throughout the 2YL [34]. Although our study design does not help identify reasons for the MOV, reasons for MOV in other studies have been shown to be multifaceted at national, sub-national, and local levels and therefore require setting-specific and adaptable mitigation strategies. Under-vaccinated children in low and middle-income countries frequently have MOV when they seek care from health facilities [36]. Commonly, a MOV occurs during immunization sessions due to upper age limits on some routine vaccinations, especially after the child’s first year of life, hindering a child’s ability to be caught up on missed vaccinations [37], [38]. An enabling policy environment that permits catch-up vaccination into the 2YL and beyond is therefore a critical aspect of a functional 2YL platform. Poor healthcare worker training on immunization schedules and insufficient use of HBR have also been shown to contribute to MOV [39], [40].

An effective catch-up vaccination strategy requires that healthcare workers request and screen HBR during every health visit [41]. Training healthcare workers to regularly screen and rigorously identify missed vaccinations is essential to having a robust system for catch-up vaccination. However, the availability of HBRs was low in our analysis of Pakistan, the Philippines, and South Africa. This is consistent with findings in other low-resource settings [42], [43], [44]. Reasons for the low HBR prevalence vary but are not restricted to household reasons. While there are systematic problems with HBR availability due to national stock-outs of vaccination cards such as reported in the Philippines and other nations in 2014–15 [45], there are also issues with the quality of data available and recorded as a result of illegible or incomplete records, loss/damage of the physical cards after exposure to harsh environments, and ineffective layout design of the card amongst others [46], [47].

Our analyses have limitations. First, non-universal HBR availability in all three countries limits the generalizability of the results within each country. Some analyses, particularly the reverse Kaplan Meier curves, require vaccination dates and can only be generalizable in settings with a high prevalence of HBR availability. However, if we had required a high HBR prevalence as inclusion criteria, most low or lower-middle-income countries would not be eligible [42], [44]. Our analyses are therefore biased toward the sub-populations with HBR availability, likely the populations with better access and utilization of services. Hence, immunization programme weaknesses identified through these analyses are likely an under-estimation, whereas programme strengths identified should be interpreted with caution. Second, there might have been valid reasons to not offer a vaccine dose, such as a contraindication. Nevertheless, such reasons tend to represent a very low proportion of cases. Third, our results of three countries cannot be generalized neither to each entire country nor globally. However, by observing similar findings in countries from three different regions, there is a likelihood that similar issues with catch-up vaccination in the 2YL are also experienced in many other countries. Lastly, our recall estimates in Table 3 might have been influenced by Supplemental Immunization Activities (SIAs). But our Kaplan-Meier analyses were based on documented dates on vaccination cards and thus unlikely to be influenced by SIA doses.

Service delivery disruptions caused by the COVID-19 pandemic have highlighted the need to build resiliency within immunization programmes to be able to recover rapidly and efficiently. Regularly screening and providing catch-up vaccinations to children through the routine immunization programme, particularly through a robust 2YL platform, are critical to pandemic recovery efforts. Opportunities during 2YL and beyond, such as child-health days and vaccination checks at sick child visits and school vaccination days, must be capitalized upon to close immunity gaps, ensure that children are protected, and reduce the risk of VPD outbreaks [34]. Strengthening the 2YL platform aligns with the Immunization Agenda 2030 (IA2030) strategic priorities on ‘Coverage and Equity’ and ‘Life course and integration,’ and contributes to IA2030′s vision to leave no one behind [48]. All three countries included in our analysis reported not having a system to monitor vaccination given after the recommended age, this limits the ability of immunization programmes to monitor children “recovered” as a result of catch-up activities [2]. As immunization systems get updated and new household surveys become available, we might be able to determine how much 2YL platforms have actually been used for catch-up vaccination efforts since the start of the pandemic and beyond. As COVID-19 vaccines become approved for younger populations, it will become even more vital to strengthen existing vaccination platforms, as well as immunization information systems, to deliver vaccinations across the life course, including for catch-up vaccination.

**Disclaimer**

The authors alone are responsible for the views expressed in this paper, and they do not necessarily represent the views, decisions, or policies of the institutions with which they are affiliated.

## Declaration of Competing Interest

The authors declare that they have no known competing financial interests or personal relationships that could have appeared to influence the work reported in this paper.

## Data Availability

We used open access DHS data sets.
