# Corrigendum to “Use of catch-up vaccinations in the second year of life (2YL) platform to close immunity gaps: A secondary DHS analysis in Pakistan, Philippines, and South Africa” [Vaccine 41(1) (2023) 61–67]

**DOI:** 10.1016/j.vaccine.2023.02.033

**Published:** 2023-03-31

**Authors:** Porcia Manandhar, Kathleen Wannemuehler, M. Carolina Danovaro-Holliday, Laura Nic Lochlainn, Stephanie Shendale, Samir V. Sodha

**Affiliations:** a Department of International Health, Johns Hopkins Bloomberg School of Public Health, Baltimore, MD, USA; b Department of Biostatistics & Medical Informatics, University of Wisconsin – Madison, WI, USA; c Department of Immunization, Vaccines and Biologicals (IVB), World Health Organization, Geneva, Switzerland

The authors regret the errors made in Fig. 2 describing MCV coverage in the published article. DTP data was mistakenly used in the figure instead of MCV coverage data. This mistake does not affect the findings of our study.

The correct figure is as follows:

**Fig. 2:**
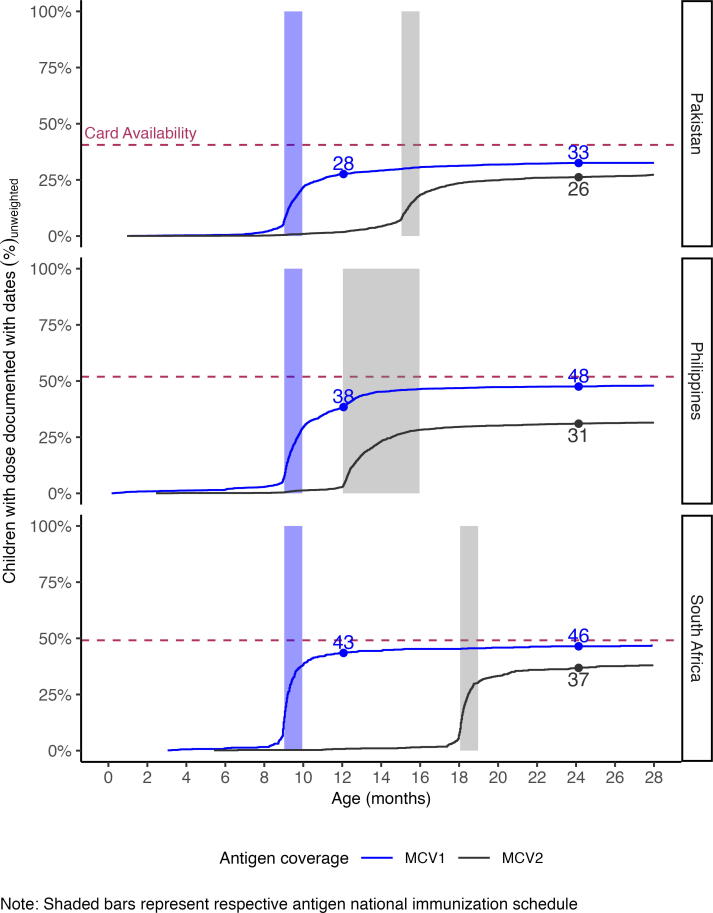
MCV Vaccination coverage by age in months amongst 24–35 month-olds in Pakistan (DHS 2017–2018), the Philippines (DHS 2017), and South Africa (DHS 2016).

The authors would like to apologise for this oversight and any inconvenience caused.

